# Association of *TIMP-1* and *COL4A4* Gene Polymorphisms with Keratoconus in an Iranian Population

**DOI:** 10.18502/jovr.v15i3.7448

**Published:** 2020-07-29

**Authors:** Davood Yari, Zohreh Ehsanbakhsh, Mohammad-Hosein Validad, Farzaneh Hasanian Langroudi

**Affiliations:** ^1^Cellular and Molecular Research Center, Zahedan University of Medical Sciences, Zahedan, Iran; ^2^Department of Clinical Biochemistry, School of Medicine, Zahedan University of Medical Sciences, Zahedan, Iran; ^3^Mashhad University of Medical Sciences, Mashhad, Iran; ^4^Shariati Hospital, Mashhad University of Medical Sciences, Mashhad, Iran; ^5^Department of Ophthalmology, Alzahra Eye Hospital, Zahedan University of Medical Sciences, Zahedan, Iran

**Keywords:** Collagen, COL4A4, Keratoconus, Polymorphism, TIMP-1

## Abstract

**Purpose:**

Keratoconus (KC) is a bilateral and noninflammatory disease, characterized by progressive thinning and anterior protrusion of the cornea and may result in severe visual impairment due to irregular astigmatism. Matrix metalloproteinases (MMP) are the main group of enzymes that degrade extracellular matrix proteins including collagens; Type IV collagen is found in the corneal stroma. MMP enzymatic activity is inhibited by tissue inhibitor of metalloproteinase-1 (TIMP-1). A decrease in TIMP-1 level is associated with the development of KC. In the present study, we investigated the impact of *COL4A4 *rs2228557 C/T and *TIMP-1* rs4898 C/T (X-chromosome) variants on the odds of KC development in a sample of Iranian population.

**Methods:**

This case–control study was conducted on 140 patients with KC and 150 healthy control subjects. We used modified methods of Nested-PCR and ARMS-PCR in combination (Nested-ARMS-PCR) and confirmed their validity with RFLP–PCR.

**Results:**

Significant differences were noticed between KC patients and healthy individuals regarding the genotype TY or T allele frequencies of rs4898 in the male subjects (OR = 0.43, 95%CI: 0.20–0.92, *P* = 0.03), whereas no significant differences were identified in the female subjects (OR = 1.07, 95%CI: 0.52–2.20, *P* = 0.85). The rs2228557, T allele was associated with KC (OR = 0.69, 95% CI: 0.50–0.97, *P* = 0.035).

**Conclusion:**

In the rs2228557 variant, T allele acts as a protective factor from the disease and decreases the risk of KC compared with the C allele. Also, in our investigation about rs4898, we found that TY genotype or T allele decreased the risk of KC compared with the C allele in males and was a protective factor for KC in our population

##  INTRODUCTION

Keratoconus (KC) is defined as a bilateral, non-inflammatory, and progressive disease characterized by conical protrusion of the cornea. This disease may result in severe visual impairment due to irregular astigmatism and stromal scarring. KC eventually affects both eyes, although the involvement is usually asymmetric. The symptoms of KC-affected patients are different depending on the stage of the disease.^[1,2,3]^ Glasses or contact lenses can provide useful vision in the early stage of the disease; nonetheless, corneal transplantation is mandatory for visual rehabilitation in 20% of the patients who are in advanced stage. Corneal thinning is considered as one of the identifying characteristics of KC. Central corneal thickness (CCT) is lower in KC patients by 75 µm as compared to normal controls.^[4,5]^

The incidence of KC is approximately 1 per 2,000, and its prevalence is 54.5 per 100,000. This disease occurs in both genders,^[6]^ with different rates among different ethnic groups.^[7]^ KC usually begins in teens, and its progression slows after the age of 30 years.^[6]^

KC is a multi-factorial disorder; environmental factors cause KC in genetically susceptible individuals. The environmental factors that may play roles in the pathogenesis of KC include eye rubbing, allergy, connective tissue dysfunction, and contact lens wear. Moreover, subjects with a family history of KC are more susceptible to this disorder.^[8]^ Using family-based linkage, several case–control studies have determined various genes that increase the odds of KC development.^[9]^ The gene candidates for KC include *LOX*,^[10]^ *VSX1*,^[11]^ *GPX-1*,^[12]^ *TGF-β1*,^[13]^ *COL4A3* and *COL4A4* ^[14]^ (polymorphism or mutation), and *TIMP-1*,^[15]^ *MMP-2*, *MMP-9* ^[16]^ (gene expression). KC is still an enigmatic disease in many aspects, including inheritance, basic pathophysiology, prevention, associated risk factors, disease development, as well as therapeutic approaches.^[17]^

Type IV collagen is only present in basement membranes and constitutes their main structural component. Collagen type IV gene, *alpha-4 (COL4A4),* is located in the region 2q35–q37 with a gene span composed of 113 kb and 48 exons.^[18,19]^ Type IV collagen is not expressed in cornea in the normal condition and its presence indicates a corneal pathology; therefore, type IV collagen can be a potential candidate in the pathogenesis of KC. In support of this theory, alterations in the expression level of collagen type IV (α-4 chains) were observed in corneas inflicted by KC.^[20,21]^ Matrix metalloproteinases (MMP) are the major expressed metalloproteases in the cornea. It has been demonstrated that the proteolytic activity of MMP increases in KC. This finding suggests that abnormal MMP activity plays a role in the pathogenesis of KC.^[16,22]^

Four types of tissue inhibitor of metalloproteinase (*TIMP1-4*) have been detected; three of which, including *TIMPs-1*, 3, and 4, are nested within an intron in the genes of synapsins. TIMP inhibits collagenases and proteoglycanase called matrix metalloproteinase. All four types of TIMPs have various biological activities apart from their metalloproteinase inhibitory activity. These biological activities include the promotion of cell proliferation, cancer promotion, regulation of angiogenesis, as well as pro- and anti-apoptotic and synaptic flexibility activities, many of which are independent of metalloprotease inhibition. *TIMP-1* is associated with synapsin 1 and its gene is located in X11p11.23–11.4 consisting of six exons. Mature *TIMP-1* is a 28.5 kDa glycoprotein that consists of 184 amino acid residues. The natural precursor contains a signal peptide of 23 residues which are cleaved throughout the protein maturation.^[23,24,25,26,27]^ *TIMP-1* suppresses angiogenesis and controls the balance in the corneal tissue by inhibiting the action of matrix metalloproteinase to protect tissues from permanent damage.^[28]^ Furthermore, it has been demonstrated that increased MMP and decreased TIMP levels are associated with the development of KC.^[29]^

*COL4A4* gene rs2228557 (F1644F) (HGVM1660028) is located in chromosome 2 exon 48, NM_000092.4 region. Several studies examined the association between *COL4A4* and KC and revealed that this gene is associated with KC; however, some other studies failed to find the same relationship in different populations.^[14,22,30,31]^

*TIMP-1* gene rs4898 (+372C/T) (HGVM6380940) is located within the intron of the synapsin gene, and single nucleotide polymorphism (SNP) is located in exon 5, the region of NM_006950.3. Some studies investigated the association of rs4898 gene polymorphism with disorders including intracerebral hemorrhage,^[32]^ systemic sclerosis,^[33]^ and severe sepsis.^[34]^

The present study aimed to evaluate the possible association of *TIMP-1* rs4898 C/T gene polymorphism and *COL4A4* rs2228557 C/T gene polymorphism with the development of KC in a sample of Iranian population.

##  METHODS

### Patients

The current retrospective case–control study was conducted at the Alzahra Eye Hospital, Zahedan University of Medical Sciences, Zahedan, Iran and recruited 140 unrelated Iranian patients with KC and 150 unrelated healthy controls. The patients were diagnosed with KC after a comprehensive ophthalmic examination using the following criteria: (1) clinical signs of KC (Munson sign, protrusion, Vogts striae, corneal thickness, scarring, Fleischer ring) and abnormal findings in corneal topography (Pentacam AXL, OCULUS INC); (2) the three quantitative videokeratographic indices used for the screening of KC were central corneal power > 47.2 D, inferior–superior value > 1.4 D, Sim-K astigmatism > 1.5 D, and skewed radial axes > 21°.^[10,35]^ Patients with other ocular diseases were excluded from the study. Controls were sex- and age-matched healthy participants who were unrelated to the patients and were selected from a geographic area similar to that of KC subjects.

The Ethics Committee of Zahedan University of Medical Sciences, Zahedan, Iran approved the study protocol and informed consent was signed by all participants. This study complied with the tenets of the Declaration of Helsinki.

### Analysis of the *TIMP-1* (rs4898), COL4A4 (rs2228557) Polymorphisms

Blood samples were collected in EDTA-containing tubes and genomic DNA was extracted from the peripheral blood leukocytes using salting out method as previously described.^[36]^ All procedures were performed under a standardized setting to avoid variation in DNA quality. SNP rs2228557 *COL4A4* was evaluated by ARMS–PCR. For the detection of rs4898 *TIMP-1*, we used the combination of Nested-polymerase chain reaction (Nested-PCR)^[37]^ and amplification refractory mutation system-PCR(ARMS-PCR)^[38]^ (Nested-ARMS-PCR). The verification of these methods was accomplished using Restriction Fragment Length Polymorphism (RFLP-PCR).^[39]^

The rs4898 location was very challengeable for ARMS-PCR; therefore, for the detection of the SNP, we used Nested or hemi-Nested-PCR primers, as mentioned previously.^[40]^ The advantages of this modification include elimination of non-specific products, protection of SNP position for the next steps, low cost, and short duration of the process.

**Figure 1:**
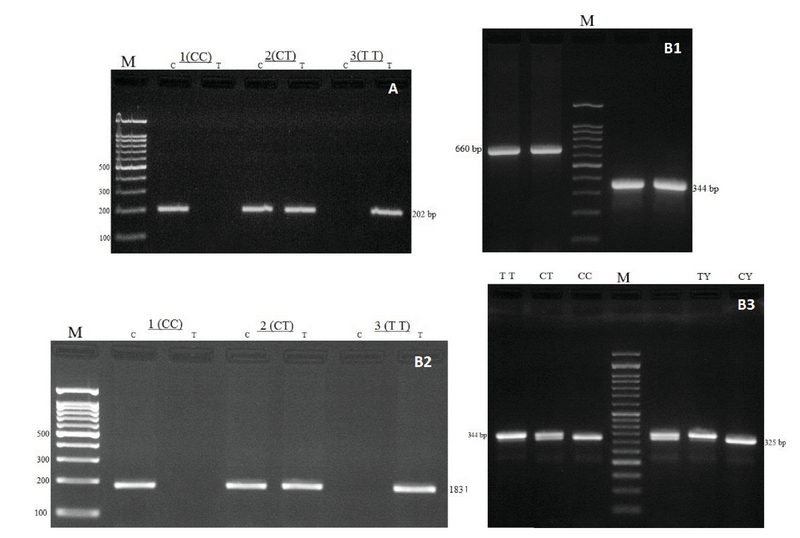
Electrophoresis pattern for the detection of SNPs in *COL4A4* rs2228557 and *TIMP-1* rs4898. **(A)** Arms-PCR products of COL4A4 rs2228557, M: DNA marker (100 bp). **(B1)** *TIMP-1* rs4898, Nested-PCR products, M: DNA marker (100bp), product size (660 and 344bp), respectively. **(B2)** ARMS-PCR products, product size (202bp), (TT, TC, CC) demonstrate genotypes. **(B3)** RFLP-PCR products, M: DNA marker (50bp), product sizes were 344bp for TT, 344bp and 325bp for TC, 325bp for CC in female, 344bp for TY and 325bp for CY in male, (TT, TC, CC) demonstrate female and (TT = TY, CC = CY) demonstrate male genotypes, (Y stands for the Y-chromosome).

PCR reactions were performed using PCR master mix (Ampliqon Taq 2x mastermix, Denmark) according to the manufacturer's instructions. For investigation of *COL4A4* (rs2228557), amplification reaction was provided in 20 μL volume including: 1 μL template DNA (∼100 ng/μL), 1 μL of each primer (10 pmol/μL), 10 μL mastermix, and 7 μL DNase-free water. The PCR conditions were set as follow: 95°C for 5 min, 30 cycles of 95°C for 30 sec, 55°C for 35 sec, 72°C for 30 sec, and a final extension at 72°C for 5 min. PCR products were detected by electrophoresis on a 2% agarose gel staining by ethidium bromide (Figure 1A).

In the first stage of study for *TIMP-1* rs4898, Nested-PCR reaction was performed in 20 μL volume including: 1 μL template DNA (∼100 ng/μL), 1 μL of each primer (10 pmol/μL), 10 μL mastermix, and 7 μL DNase-free water. The PCR conditions were set as follow: 95°C for 5 min, 30 cycles of 95°C for 30 sec, 64°C for 40 sec, 72°C for 30 sec, and a final extension at 72°C for 5 min. In the second phase, the PCR product obtained from the first stage (660 bp) was used as the template and diluted 1:50. Primers for ARMS-PCR were designed to detect the SNP (Table 1). In this step, ARMS-PCR reaction was performed in 20 μL volume including: 1 μL template (1:50 dilution), 1 μL of each primer (10 pmol/μL), 10 μL mastermix, and 7 μL DNase-free water. The PCR conditions were set as follow: 95°C for 5 min, 17 cycles of 95°C for 30 sec, 56°C for 30 sec, 72°C for 30 sec, and a final extension at 72°C for 5 min. PCR products were detected by electrophoresis on a 2% agarose gel staining by ethidium bromide (202bp product). Consequently, SNP (rs4898) *TIMP-1* was successfully detected with the combination of two methods (Nested-PCR and ARMS-PCR) (Figures B1 & B2).

**Table 1:** The list of primers and methods used for detection of Single Nucleotide Polymorphisms (SNPs) *TIMP-1* rs4898 and *COL4A4* rs2228557


**TIMP-1(rs4898) T/C**		**Primers(5'-3')**	**Product size**
**Stage 1Nested-PCR**	F	TGGGGACACCAGAAGTCAAC	660 bp
		R		TAAGCTCAGGCTGTTCCAGG	
**Stage 2ARMS-PCR**	F Common	AGGCTCTGATGAGAATGGTCCCA	202 bp
		R (C allele)		CAGATTGTTCCAGGGAGCCAAG	
		R (T allele)		CAGATTGTTCCAGGGAGCCAAA	
**Stage 3RFLP-PCR**	F	CCGCCATGGAGAGTGTCTGC	344 bp
		R*		AGGCTGTTCCAGGGAGTCGC	
**COL4A4(rs2228557 ) C/T**		
**ARMS-PCR**	F Common	TGTCTGAGCCCTAATTCTCT	183 bp
		R (C allele)		GAGCCAGAAGCTATACTTATTTGAG	
		R (T allele)		GAGCCAGAAGCTATACTTATTTGAA	
F, forward; R, reverse; R*, altered reverse			

**Table 2:** Genotype and allelic frequencies of *COL4A4* rs2228557 and *TIMP-1* rs4898 polymorphisms between keratoconus (KC) patients and healthy controls.


**Variants**	**KC Patients n (%)**	**Controls n (%)**	*OR (95% CI)	**** ***P*** **-value**
**rs2228557,COL4A4**		
**CC**	67 (47.8)	61 (40.7)	Ref.	—
**CT**	39 (27.9)	37 (24.7)	0.96 (0.54–1.69)	0.887
**TT**	34 (24.3)	52 (34.6)	0.59 (0.34–1.03)	0.067
**Allele**		
**C**	173 (61.8)	159 (53)	Ref.	—
**T**	107 (38.2)	141 (47)	0.69 (0.50–0.97)	0.035
**rs4898,TIMP-1**	Male	Male	
**CY**	46 (75.4)	37 (57)	Ref.	—
**TY**	15 (24.6)	28 (43)	0.43 (0.20–0.92)	0.038
****	Female	Female	
**CC**	28 (47.5)	31 (52.5)	Ref.	—
**CT**	29 (49.2)	30 (50.8)	1.01 (0.46–2.19)	0.97
**TT**	22 (47.8)	24 (52.2)	1.07 (0.52–2.20)	0.854
**Allele**		
**C**	85 (53.8)	92 (54.1)	Ref.	—
**T**	73 (46.2)	78 (45.9)	1.01 (0.66–1.56)	0.953
Ref., reference; OR, odds ratio; CI, confidence interval; n, number *Adjusted for sex and age. (Y states for the Y-chromosome)				

**Table 3:** Correlation of clinical and keratometric parameters with COL4A4 (rs2228557) and TIMP-1(rs4898) in keratoconus patients.


**Parameters evaluated**	**Patients n (%)**	**rs2228557**	**** ***P*** **-value**	**rs4898**	**** ***P*** **-value**
		**Male**	**Female**
**KC ocular**			
**OD**	42 (30.0)	0.25	0.39	0.4
**OS**	36 (25.7)					
**OU**	62 (44.3)					
**Level of KC**			
**KK 1**	33 (23.6)	0.81	0.014	0.97
**KK 2**	45 (32.1)					
**KK 3**	62 (44.3)					
**CXL**			
**OD**	39 (27.9)	0.58	0.71	0.37
**OS**	40 (28.6)					
**OU**	42 (30.0)					
**Candidate**	19 (13.6)					
Correlation of clinical and keratometric parameters with *COL4A4* (rs2228557) and *TIMP-1* (rs4898) in keratoconus patients. KC, keratoconus; OD, right eye; OS, left eye; OU, both eyes; CXL, cross-linking surgery KK1, 2, 3 are phenotyping classification and show the level progress of keratoconus disease in patients			

RFLP-PCR was applied to validate the method and results of screening of rs4898 polymorphism in *TIMP-1*. As the original sequence of the region surrounding the polymorphism does not make a restriction enzyme site, a site-directed mutagenesis PCR primer (R*) was designed. This primer differs from the referent sequence in two bases and lies close to the polymorphic spot to alter the sequence and provide a restriction enzyme site at product. Thus, the original sequence of the region is TT(C) GTGG, while our PCR product was in fact TT(C) GCGA. The C-variant is a palindrome which forms a site for the Bsp68I (NruI) restriction enzyme (Thermo scientific).^[40]^

Initially, we amplified PCR product (660bp) using the abovementioned conditions. For alteration in the restriction enzyme site, secondary primers were added to template 1:50 dilution of first stage using the following condition: 1 μL template (1:50 dilution), 1 μL of each primer (10 pmol/μL), 10 μL mastermix, and 7 μL DNase-free water. The PCR conditions were set as follow: 95°C for 5 min, 30 cycles of 95°C for 30 sec, 63°C for 30 sec, 72°C for 30 sec, and a final extension at 72°C for 5 min. PCR products were detected by electrophoresis on a 2% agarose gel with ethidium bromide (344bp product). For optimal results, PCR products were digested at 37°C for 5 h according to the manufacturer's instruction. The restriction of the C-allele PCR product resulted in 325bp and 19bp digest products, whereas the T allele (wild type) products remained unrestricted (Figure B3).

### Statistical analysis

Statistical analyses were performed using the SPSS software version 19.0 (SPSS Inc., Chicago IL, USA). Frequencies were compared between the study groups using the Chi-square test. Association of gene polymorphisms with KC was investigated and compared between the groups using logistic regression analysis, estimation of odds ratio (OR), and 95% confidence intervals (CI), respectively. A *p*-value < 0.05 was regarded as statistically significant.

##  RESULTS 

A total of 140 patients (61 male and 79 female subjects), aged 28 ± 12.5 years, included the KC group. The control group consisted of 150 healthy controls (65 male and 85 female subjects), aged 29.8 ± 15.6 years.

There was no significant difference between the two groups regarding the age and gender (*P* = 0.20).

The *Col4A4* rs2228557 C/T variant, T allele was associated with a decrease in the risk of KC development (OR = 0.69, 95% CI: 0.50–0.97, *P* = 0.035), as compared to C allele. Our results indicated that TT was not associated with KC as compared to CC (OR = 0.59, 95% CI = 0.34–1.03, *P* = 0.067) (Table 2).

Table 2 demonstrates the genotype and allelic frequencies of *TIMP-1 *(rs4898) gene polymorphism in each study group. Since *TIMP-1* is an X-linked gene, the results were compared in male and female subjects separately. This analysis demonstrated that TY genotype or T allele decreased the risk of KC in male subjects as compared to the C allele (OR = 0.43, 95% CI: 0.20–0.92, *P* = 0.03). However, no significant association was found between TT genotype (OR = 1.07, 95% CI: 0.52–2.20, *P* = 0.854) or T allele (OR = 1.01, 95%CI: 0.66–1.56, *P* = 0.95) and KC in female subjects.

Table 3 illustrates the associations of *COL4A4* (rs2228557) and *TIMP-1* (rs4898) with KC severity. This polymorphism *TIMP1* (rs4898 T/C) located at X chromosome exists in two alleles and their combination results in five possible genotypes. Because men lack a second X-chromosome, the possible genotypes are CY and TY hemizygotes (Y states for the Y-chromosome). As for women, there are CC and TT homozygotes and CT heterozygotes (Table 2). In fact, men just have allele and women have genotype and allele. For this reasons, calculation of *TIMP1* (rs4898) distinctly separated in men and women was done. So, results in men and women can be different.

##  DISCUSSION

Keratoconus is an eye disorder characterized by bilateral, asymmetrical, noninflammatory, and progressive thinning of the cornea. The shape of cornea progressively alters from the normal round shape to a cone shaped one. Although the etiology of KC is not clear, defective cross-linking between adjacent collagen fibers may play an important role in its pathogenesis.^[41]^ SNPs and gene variants suggest an intricate etiology or the convergence of multiple disease pathways.^[42]^

Biochemical investigations have suggested that the amount of collagen fibers decrease in KC. Also, the weight of KC corneas was found to be reduced; therefore, it can be assumed that collagenase enzymes might be involved.^[43]^

In the current study, we investigated the impact of *COL4A4* and *TIMP-1* variants on the risk of KC development in a sample of Iranian population. Our results showed that the T allele reduced the risk of disease development, as compared to the C allele. Results in the distribution of genotypes (CC, CT, TT) in rs2228557 of the *COL4A4* gene between KC patients and controls in the Stabuc-Silih et al's study were different from our results.^[14]^ Similar to our findings, Kokolakis et al demonstrated that the TT genotype was significantly over-represented in healthy individuals and suggested a protective role for this genotype in the KC development.^[31]^

The level of *TIMP-1* significantly decreases in KC as compared to normal corneas. Given the fact that KC is not associated with extensive scarring or inflammatory infiltrates, substantial degradation should occur in the extracellular matrix. A decreased level of TIMP-1 increases gelatinase and collagenase activities and apoptosis which are characteristic phenomena in KC. Decreases in *TIMP-1* might play a role in matrix degradation which is a characteristic feature of KC.^[15,44]^ Furthermore, it has been recognized that increased MMP and decreased *TIMP-1* levels are associated with the development of KC.^[29]^

The *TIMP-1* gene is located in Xp11.3–p11.23 and has three types of polymorphism including *TIMP-1 *(19 C/T) in the 5'-UTR, *TIMP-1* (261 C/T) in exon 4, and *TIMP-1* (372 T/C) (rs4898) in exon 5. The *TIMP-1 *(rs4898) polymorphism is an important site which has been reported in other studies. This variation does not result in changes in the amino acid sequence (F124F). This polymorphism exists in two alleles and their combination results in five possible genotypes. Because male subjects lack a second X-chromosome, the possible genotypes are CY and TY hemizygotes. In female subjects, however, there are CC and TT homozygotes and CT heterozygote genotypes.^[40]^

Our data suggest that C or T allele is associated with *TIMP-1 *(rs4898) polymorphism in patients or controls. The C allele of the 372T/C polymorphism was more frequently found in female than male controls.^[45]^ However, in other studies, the C allele was detected more frequently in male patients with an abdominal aortic aneurysm.^[46]^ Meijer et al investigated the male subjects with inflammatory bowel disease carrying *TIMP-1* (rs4898) T allele. They reported lower levels of TIMP-1 in surgically resected inflamed tissue, as compared to C allele carriers.^[47]^ In addition, Indelicato et al reported that *TIMP-1* (rs4898) C allele frequency increased in males but not females with systemic sclerosis, as compared to healthy individuals.^[48]^ Along the same lines, Wei et al revealed that C allele carriers of *TIMP-1* (rs4898) run a greater risk of developing ankylosing spondylitis disease.^[49]^ Furthermore, it was found that among cirrhotic patients, males with *TIMP-1* (372C/T) T allele developed cirrhosis at a younger age.^[50]^

Our findings indicated that *TIMP-1 *(rs4898) was associated with the clinical characteristics of KC only in our male sample population. Nevertheless, the analysis of genotype and allele frequencies revealed no significant differences in female patients as compared to female controls. The T allele decreased the risk of KC, as compared to the C allele in males which can be attributed to the location of *TIMP-1* gene at Xp11.3–p11.23. Males only have one X-chromosome, and the functional difference of genetic polymorphism of *TIMP-1* (rs4898) appears more obvious due to the lack of heterozygotes. We cannot compare our results with the literature because no previous study has evaluated the correlation between the *TIMP-1* variants and the risk of KC development.

In conclusion, our study showed that in the *COL4A4* rs2228557 C/T variant, the T allele acts as a protective factor against the disease and decreases the risk of KC. In addition, *TIMP-1* rs4898 C/T the TY genotype or T allele in males can decrease the risk of KC in comparison with the C allele. Further studies with a larger sample size and different ethnicities are required to confirm these findings.

##  Financial Support and Sponsorship

This study was supported by a dissertation grant (M.Sc. thesis of Davood Yari, NO. 6077) from the Deputy for Research, Zahedan University of Medical Sciences.

##  Conflicts of Interests

There are no conflicts of interest.
